# Therapeutic Aspects of Circadian Rhythms

**DOI:** 10.3390/biom13081169

**Published:** 2023-07-27

**Authors:** James C. Walton, Randy J. Nelson

**Affiliations:** Department of Neuroscience, Rockefeller Neuroscience Institute, West Virginia University, Morgantown, WV 26506, USA; randy.nelson@hsc.wvu.edu

Circadian rhythms are ubiquitous endogenous rhythms with a period of approximately twenty-four hours. These fundamental biological rhythms evolved to align behavioral and physiological rhythms with natural light-dark cycles to optimize health, reproduction, and survival. Although circadian rhythms underlie virtually all biological processes it is quite surprising that most fields of study, from basic and preclinical research to pharmacology and medicine, largely ignore time of day as an important biological variable [1,2]. Furthermore, disruption or misalignment of internal rhythms with the external environment is detrimental to health and well-being [3,4]. To begin to address some of these gaps in the literature, we edited this Special Issue of *Biomolecules*, entitled “Therapeutic Aspects of Circadian Rhythms”. This issue contains three original research reports and five reviews that explore molecules and processes that transduce the environmental signal (light) into a biological signal, how time is kept in a biological system, and the negative outcomes of misalignment of internal rhythms with external rhythms when that system is perturbed [5,6,7,8,9,10,11,12].

The biomolecule melatonin is produced in multiple tissues and was first identified as a hormone excreted from the pineal gland at nighttime, as such it is considered the “hormone of darkness”. Melatonin has other functions however, and the signal transduction pathway that entrains circadian rhythms to external light dark cycles begins in the eye. The review by Martínez-Águila and colleagues [9] describes the role of ocular-synthesized melatonin and the interaction of circadian rhythms and clock genes in ocular physiology and diseases. The authors provide clear evidence from studies of intraocular pressure that, in common with most biological processes, it will be critical for biomedical researchers and physicians to monitor eye conditions across the day to provide the best diagnoses and treatment.

In addition to diseases of the eye, there is growing evidence that other disorders have a circadian component. In this issue, we present several articles that focus on circadian rhythms in cardiovascular health and disease. Mikulska and coauthors [8] examine the relationships between the core clock genes CRY1 and BMAL and blood pressure in women and reported that CRY1 may be useful as a predictive diagnostic for risk of hypertension. From a broader perspective, a review from our group [11] takes a deeper look into the influence of sex, time of day, and circadian rhythms on overall cardiovascular health. We review cardiovascular rhythms and pathologies associated with disruption of these circadian rhythms, and how biological sex influences these outcomes. We conclude that in the clinical literature for cardiovascular health, time of day is largely unrecorded and unreported, despite the strong evidence that it is a critical factor. The paucity of studies that consider circadian rhythms in their design is striking, and we strongly urge clinicians to incorporate this variable into their reports and experimental designs. Bridging these two papers on cardiovascular disease, Zhang and colleagues [5] review circadian rhythms in blood pressure and renal disease and how disrupted rhythms may exacerbate kidney diseases. They propose the potential benefits of chronotherapy for BP maintenance and kidney disease and discuss the gaps in knowledge of the molecular links between kidney disease and circadian clock genes.

Circadian clock genes are not only linked to vascular and renal diseases, they are also linked to neurodegenerative diseases and altered cognition. A review by Maiese [10] in this special issue explores the interaction of circadian clock gene pathways with dementia and other cognitive diseases. Focusing on evidence from pathways involved in autophagy, several circadian clock genes central to this pathway are proposed as therapeutic targets to intervene in neurodegenerative diseases and age-related cognitive decline. More direct evidence of the effects on brain function of circadian disruption by light at night come from a study in this issue exploring the exposure of zebra finches to intensities of light pollution at night found outdoors in urban areas of the world (1.5–5 lux). These low levels of light exposure altered neuronal recruitment and turnover in brain regions involved in social communication and cognition in a sex-specific and region-specific manner [7]. This study raises some important points for readers of this special edition, not only are there sex differences in circadian rhythms [13], disrupted rhythms by light at night occur in both indoor and outdoor environments. Furthermore, as more lines of research converge to assess the role of circadian rhythms in health and disease and look to identify the molecular underpinnings of the connections between these factors, an issue with standard methodology of gene expression analysis becomes readily apparent; what are the appropriate reference genes for circadian studies? Quantifying gene expression with qPCR requires normalization to reference genes, however many of the “standard” reference genes are not stable across the day and vary expression patterns dependent upon tissue, species, and strain [14]. Thus, to be able to compare circadian expression patterns across studies, each tissue or organ necessarily needs to have a set of stable reference genes identified. Toward this end, Szczepkowska and coauthors identify a stable set of reference genes for circadian expression analyses of brain microvessels and choroid plexus in rats [6].

Individual variation in circadian rhythmicity manifests behaviorally as a chronotype, meaning that some have phase advanced daily rhythms of sleep and waking earlier in the day, morning people or larks, whereas others have a delayed rhythms and are evening people or night owls, the remainder fall somewhere in between. Montaruli and colleagues [12] explore the effects of chronotype on susceptibility to, and the interaction with, neurodegenerative and cardiovascular diseases and how chronotype can affect quality of life when rhythms are disrupted. Assessment and consideration of an individual's chronotype will become a critical biological factor as the fields of personalized medicine and chronotherapeutics advance and necessarily converge.

Taken together, we hope you find this special issue of *Biomolecules* informative in highlighting the critical importance of time of day and circadian rhythms in health and disease. Good circadian hygiene (avoiding disrupted circadian rhythms), facilitated by brightly illuminated days and dark nights, promotes the most robust health and well-being. It is also critical to understand the daily rhythmicity of the processes underlying diseases of all types, and then incorporating chronotherapeutics and chronotype into personalized treatment plans are the next critical steps forward in approaches to successful interventions.
